# Plasma Metabolome Profiling Identifies Metabolic Subtypes of Pancreatic Ductal Adenocarcinoma

**DOI:** 10.3390/cells10071821

**Published:** 2021-07-19

**Authors:** Ujjwal Mukund Mahajan, Ahmed Alnatsha, Qi Li, Bettina Oehrle, Frank-Ulrich Weiss, Matthias Sendler, Marius Distler, Waldemar Uhl, Tim Fahlbusch, Elisabetta Goni, Georg Beyer, Ansgar Chromik, Markus Bahra, Fritz Klein, Christian Pilarsky, Robert Grützmann, Markus M. Lerch, Kirsten Lauber, Nicole Christiansen, Beate Kamlage, Ivonne Regel, Julia Mayerle

**Affiliations:** 1 Department of Medicine II, University Hospital, LMU Munich, 81377 Munich, Germany; ahmed.alnatsha@med.uni-muenchen.de (A.A.); lllqq07@gmail.com (Q.L.); bettina.oehrle@med.uni-muenchen.de (B.O.); elisabetta.goni@med.uni-muenchen.de (E.G.); georg.beyer@med.uni-muenchen.de (G.B.); ivonne.regel@med.uni-muenchen.de (I.R.); julia.mayerle@med.uni-muenchen.de (J.M.); 2 Department of Medicine A, University Medicine Greifswald, 17475 Greifswald, Germany; ulrich.weiss@uni-greifswald.de (F.-U.W.); matthias.sendler@uni-greifswald.de (M.S.); 3 Department for Visceral, Thoracic and Vascular Surgery, University Hospital, Technical University Dresden, 01307 Dresden, Germany; marius.distler@uniklinikum-dresden.de; 4 Department of General and Visceral Surgery, Katholisches Klinikum Bochum, 44791 Bochum, Germany; waldemar.uhl@ruhr-uni-bochum.de (W.U.); tim.fahlbusch@klinikum-bochum.de (T.F.); 5 Department of General and Visceral Surgery, Asklepios Klinikum Hamburg, 21075 Hamburg, Germany; a.chromik@asklepios.com; 6 Zentrum für Onkologische Oberbauchchirurgie und Robotik, Krankenhaus Waldfriede, 14163 Berlin, Germany; m.bahra@waldfriede.de; 7 Department of General, Visceral and Transplantation Surgery, Charité, Campus Virchow Klinikum, 13353 Berlin, Germany; fritz_klein@hotmail.com; 8 Department of Surgery, Erlangen University Hospital, 91054 Erlangen, Germany; christian.pilarsky@uk-erlangen.de (C.P.); robert.gruetzmann@uk-erlangen.de (R.G.); 9 University Hospital, LMU Munich, 81377 Munich, Germany; markus.lerch@med.uni-muenchen.de; 10 Department of Radiation Oncology, LMU Munich, 81377 Munich, Germany; kirsten.lauber@med.uni-muenchen.de; 11 TrinamiX GmbH, 67063 Ludwigshafen am Rhein, Germany; nicole.christiansen@trinamix.de; 12 Metanomics-Health GmbH, 10589 Berlin, Germany; beate.kamlage@web.de

**Keywords:** pancreatic ductal adenocarcinoma, complex lipids, sphingolipids, metabolic subtypes

## Abstract

Pancreatic ductal adenocarcinoma (PDAC) is one of the deadliest cancers. Developing biomarkers for early detection and chemotherapeutic response prediction is crucial to improve the dismal prognosis of PDAC patients. However, molecular cancer signatures based on transcriptome analysis do not reflect intratumoral heterogeneity. To explore a more accurate stratification of PDAC phenotypes in an easily accessible matrix, plasma metabolome analysis using MxP^®^ Global Profiling and MxP^®^ Lipidomics was performed in 361 PDAC patients. We identified three metabolic PDAC subtypes associated with distinct complex lipid patterns. Subtype 1 was associated with reduced ceramide levels and a strong enrichment of triacylglycerols. Subtype 2 demonstrated increased abundance of ceramides, sphingomyelin and other complex sphingolipids, whereas subtype 3 showed decreased levels of sphingolipid metabolites in plasma. Pathway enrichment analysis revealed that sphingolipid-related pathways differ most among subtypes. Weighted correlation network analysis (WGCNA) implied PDAC subtypes differed in their metabolic programs. Interestingly, a reduced expression among related pathway genes in tumor tissue was associated with the lowest survival rate. However, our metabolic PDAC subtypes did not show any correlation to the described molecular PDAC subtypes. Our findings pave the way for further studies investigating sphingolipids metabolisms in PDAC.

## 1. Introduction

Patients diagnosed with pancreatic ductal adenocarcinoma (PDAC) suffer from one of the lowest 5-year relative survival rates of 10% among all cancers, while incidence is rising by approximately 1% per year [1]. Once established the tumor grows aggressively and often without specific symptoms. Hence, the disease is detected at locally advanced or metastasized tumor stages, when surgical intervention is no longer an option [2]. Standard-of-care treatment options rely on chemotherapeutics, but the therapies are barely efficient and far away from cure. PDAC patients suffer from therapy resistance and low treatment response rates [3]. The development of diagnostic biomarkers for early cancer detection and targeted therapies is a major goal for improving the dismal prognosis for PDAC patients. Recent transcriptome approaches uncovered two to four molecular tumor signatures [4,5,6,7,8], with a consensus of a classical and basal-like PDAC subtypes [9,10]. The studies used whole tissue or laser capture microdissected (LCM) material from resected tumors or from needle biopsies of advanced cases. Examining clinical features, the classical PDAC subtype is associated with a better overall survival rate and is found more frequently in stage I/II PDAC samples, whereas the basal-like subtype is associated with a more aggressive phenotype, culminating in a worse prognosis and, in part, in chemotherapy resistance [4]. In contrast, a study from Moffitt et al. indicated that the basal-like subtype responded better to adjuvant chemotherapy [5]. Several initiatives tried to clarify the conflicting results and investigated whether molecular signatures or other biomarkers are able to predict chemotherapy response [10]. However, novel single-cell RNA sequencing data of human PDAC samples revealed intratumoral molecular heterogeneity, making prediction even more complex. A study from Chan-Seng-Yue et al. demonstrated that classical and basal-like subtypes existed, juxtaposing most of their analyzed PDAC tissue samples [8].

To overcome a misleading classification because of intratumoral heterogeneity, the analysis of blood biomarkers, particularly of metabolites, might reveal a more accurate stratification of PDAC phenotypes. Moreover, plasma is an easily available matrix for analysis without the disadvantage of putting a patient with a dismal prognosis at risk of complications from a tissue biopsy or resection. Due to high proliferation rates and the competition for nutrition and oxygen, tumor cells utilize alternative metabolic pathways to cover their elevated energy consumption [11]. Thus, metabolic changes should be easily detectable in blood samples. Indeed, in our own previous MxP^®^ broad profiling approach, we assessed metabolites in plasma and serum samples from PDAC and chronic pancreatitis (CP) patients and uncovered a metabolite biomarker signature that is able to distinguish between PDAC and CP with high sensitivity and specificity [12]. We received remarkable results, given the fact that the cohorts showed high variations in lifestyle factors. Other than amino acids, our metabolite signature contained complex lipids and CA19-9, which is currently the only PDAC biomarker used in clinical practice [12]. However, the implications of metabolic alterations within tumor cell subpopulations are poorly understood. A first comprehensive metabolic profiling of pancreatic cancer cell lines uncovered three metabolic tumor cell subtypes [13]. The low proliferation subtype exhibited low amino acid and carbohydrate levels, the glycolytic subtype revealed an enrichment of glycolysis and serine pathway components and the lipogenic subtype showed a high abundance of different lipid metabolites [13]. Interestingly, the glycolytic and lipogenic subtype were associated with the previously identified molecular quasimesenchymal/basal-like and classical subtypes, respectively [13]. Based on transcriptome data of glycolytic and cholesterol pathway genes, PDAC patients with a glycolytic phenotype showed a poor prognosis and a moderate correlation with increased KRAS and MYC gene expression levels [14]. However, targeted approaches, which focus predominantly on tumor cells and predefined metabolic pathways, might not display the full picture of complex metabolic alterations. In the present study, we used plasma metabolome profiles of 361 PDAC patients who we recruited previously to identify blood-derived biomarkers for tumor diagnosis [12]. In our comprehensive approach, we identified different PDAC subtypes based on plasma metabolite levels. The metabolic rewiring of tumor cells, which is reflected in the plasma of PDAC patients, provides great opportunities in defining cancer characteristics and clinically relevant PDAC subtypes.

## 2. Materials and Methods

### 2.1. Patients’ Recruitment and Sample Collection

The original study [12] recruited patients with pathologically confirmed pancreatic ductal adenocarcinoma (PDAC) and was performed to identify metabolic biomarkers for cancer diagnosis according to the Reporting Recommendations for Tumor Marker Prognostic Studies (REMARK) guidelines [15]. All patients gave their written informed consent and the local ethics review boards approved the protocol at all participating centers (Az. 110/99). For the present study, we included and re-evaluated 361 patients from three prospective study phases (ID, VD1 and VD2). The identification (ID) and validation cohort 1 (VD1) were described previously [12]; validation cohort 2 (VD2) is unpublished so far. Table S1 provides a detailed summary on the cohort and of patients’ characteristics. Before initiation of cancer treatment and after overnight fasting, EDTA-blood samples were collected and processed for plasma isolation, as described previously [12].

### 2.2. Metabolite Profiling

**Figure 1:**
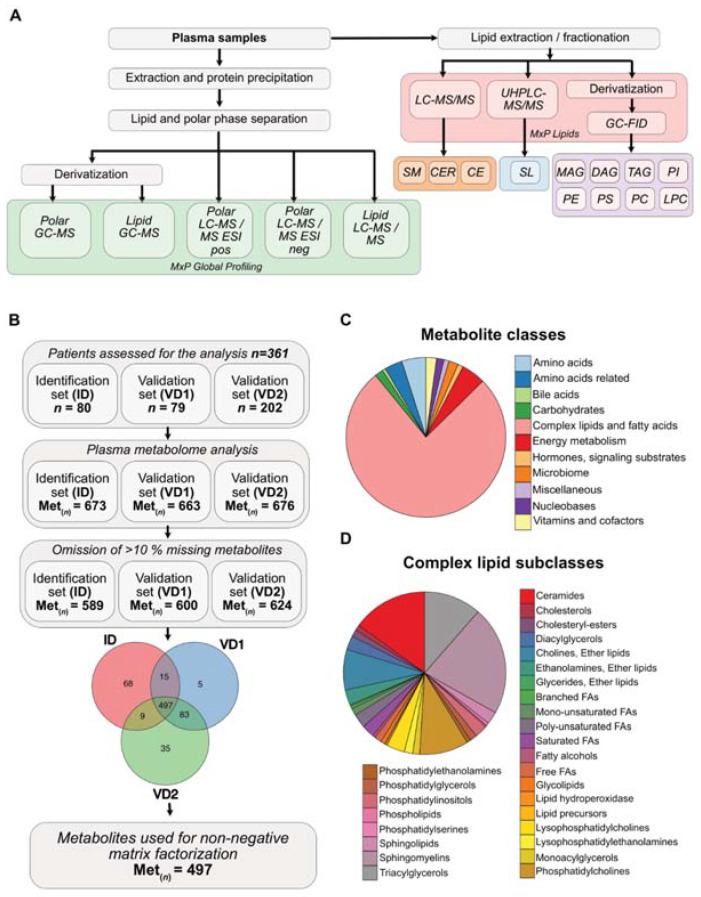
Plasma metabolome analysis of the PDAC disease cohort: (**A**) schematic representation of workflow of plasma metabolome analysis using MxP^®^ Global Profiling and MxP^®^ Lipids. GC: gas chrmoatography; MS: mass spectrospectry; ESI: electrospray ionization; UHPLC: ultra-high performance liquid chromatography; FID: flame ionization detector; SM: sphingomyelins; CER: ceramides; CE: cholesterylesters; SL: sphingilipids/sphingoids; MAG: monoacylglycerols; DAG: diacylglycerols; TAG: triacylglycerols; PI: phosphotidylinositols; PE: phosphatidylethanolamines; PS: phosphatidylserines; PC: phosphatidylcholines; LPC: lysophosphatidylcholines. (**B**) CONSORT diagram of patients recruited for the metabolic analysis and downstream metabolite selection. (**C**) Pie chart of distribution of detected metabolite classes. (**D**) Pie chart of distribution of detected complex lipid subclasses.

The plasma metabolome analysis was conducted by MxP^®^ Global Profiling (Metanomics-Health GmbH, Berlin, Germany) and an MxP^®^ Lipids (Metanomics-Health GmbH, Berlin, Germany) (Figure 1A). For the global profiling, two types of mass spectrometry analyses were applied, namely gas chromatography-mass spectrometry (GC-MS; Agilent 6890 GC coupled to an Agilent 5973 MS System, Agilent, Waldbronn, Germany) and liquid chromatography-MS/MS (LC-MS/MS; Agilent 1100 HPLC-System, Agilent, Waldbronn, Germany, coupled to an Applied Biosystems API4000 MS/MS-System, Applied Biosystems, Darmstadt, Germany). For lipid profiling, the lipids were fractionated [16] and analyzed by LC-MS/MS using electrospray ionization (ESI) and atmospheric pressure chemical ionization (APCI), with detection of specific multiple reaction monitoring (MRM) transitions for cholesterol esters (CE), sphingomyelins (SM) and ceramides (CER), respectively. Other lipid classes were measured by cas chromatography flame ionization detection (GC-FID). Sphingolipids were determined semi-quantitatively by ultra high-performance liquid chromatography (UHPLC)-MS/MS. The sample preparation for global profiling and lipids is described in detail in [12].

### 2.3. Data Normalization and Quantification of Metabolite Levels

Metabolite profiling based on a semi-quantitative analytical platform results in relative metabolite levels to a defined reference. The reference is a metabolite pool generated from aliquots from all original study samples, including among other healthy controls and PDAC patients. Four replicates of the reference pool were measured within each analytical batch that comprised a total of 24 samples (20 patient samples plus the 4 reference replicates). Since all samples were processed in different analytical batches, two different reference samples were run in parallel throughout the whole process to allow an alignment of different analytical batches. For the semi-quantitative assessment, each metabolite from each sample was normalized against the median of the corresponding metabolite in the reference pool samples within each analytical batch to provide pool-normalized ratios. This process step compensated for inter- and intra-instrumental variation, i.e., variability that occurs when different analytical batches are analyzed by different devices. To allow further quantification, an MxPool™ (a large pool of commercial human EDTA plasma from healthy donors suited for alignment of MxP^®^ studies; Metanomics-Health GmbH, Berlin, Germany) was analyzed, with 12 replicated samples. Hence, the semi-quantitative data were normalized to the median of MxPool™ samples containing more than 2500 different metabolites of known concentrations. This corresponds to a single-point calibration using multiple aliquots of the same level. As the calibration samples are of the same matrix as the project samples, they account best for all sequence-to-sequence deviations that may occur within sample preparation and measurement. Furthermore, metabolite levels of the pooled sample are representative of metabolite levels of the study, leading to the most accurate semi-quantitative values. Moreover, the slope of each metabolite signal, including unknown analytes, relative to sample amount was determined during the method development. Only metabolites with a slope justifying a single-point calibration approach were considered for the present study. A rigorous quality control was performed on peak, analyte and sample levels, and has been described previously [12,17].

### 2.4. Imputation, Scaling and Bioinformatics Applications

Overall, only metabolites with less than 10% of missing values per study phase were considered for further analyses (Figure 1B). All metabolite profiling data were log10-transformed to achieve an approximately normal distribution. The log10-transformed, auto-scaled and median imputed ratios were used for further bioinformatic applications.

To detect metabolic subtypes, non-negative matrix factorization (NMF) was used. The NMF algorithm was implemented in the R package NMF [18], and was run with a maximum iteration of 100. For each k value between 3 and 6, 1000 replicates with different random seeds were executed, and the replicate with the lowest mean-squared error and cophenetic coefficient was retained for further analysis. The final model was selected based on the lowest cophenetic coefficient for given K and NMF methods. Based on silhouette scores, the relative change between k values and the proportion of ambiguous clustering score, three main clusters were identified.

Next, weighted gene co-expression network analysis (WGCNA) was used to generate metabolic programs (MPs) per metabolic subtype [19]. To determine the enrichment of differentially expressed metabolites in modules generated by WGCNA, module metabolite enrichment was then determined and mapped using KEGG pathway analysis. Only modules showing both significant enrichment and significant metabolite expression/module correlations were considered significant.

Furthermore, ontology and pathway enrichment analysis was performed using R package, “KEGGREST” [20]. Differential expression was run in pairwise fashion across the different subtypes, as well as hybrid versus the rest. Pathway analysis of significantly enriched metabolites was performed as a differential abundance score (DAS). The DAS captures the tendency for a pathway to have increased levels of metabolites relative to a control group. The DAS was calculated as:

$$\mathrm{DAS}\;=\frac{n\left(\mathrm{metabolites}↑\right)\;-\;n\left(\mathrm{metabolites}↓\right)}{n\left(\mathrm{metabolites}↑\right)\;+\;n\left(\mathrm{metabolites}↓\right)}$$

Thus, a DAS of 1 depicts all metabolites associated with a pathway that is upregulated, whereas a DAS of −1 represents all metabolites associated with a pathway that is downregulated. The R package ‘FELLA’ [21] was also used to identify significant sub-networks of differentially expressed metabolites.

TCGA-PAAD transcriptome data counts were accessed using R package, “TCGAbiolinks” [22]. Subsets of genes associated with metabolic subtypes were analyzed for consensus clustering to demark metabolic subtypes. The date of diagnosis and the date and cause of death were obtained from the phenotype data of TCGA-PAAD. Median survival was estimated using the Kaplan-Meier method and the difference was tested using the log-rank test.

### 2.5. Statistics

All analyses were performed in R (version R-4.0.4, https://www.r-project.org/) and R-studio (version 1.3.9.59; R-studio, Boston, USA). No unique code was developed for this study. R scripts or functions used are outlined under https://github.com/mayerlelab/metabolicSubtypes.git and are available upon request. *p*-values of < 0.05 were considered statistically significant.

## 3. Results

### 3.1. Metabolite Composition of Blood Plasma Samples of PDAC Patients

The previously described, molecular PDAC subtypes rely on transcriptome data of single tumor samples, which do not reflect tumor cell heterogeneity and the co-existence of more than one molecular subtype in the tumor [10]. However, the measurement of metabolites in the blood of cancer patients might depict the overall metabolic reprogramming of the tumor, independent of intratumoral heterogeneity. Thus, we investigated metabolic plasma profiles of 361 PDAC patients. We re-evaluated a previously generated plasma metabolome and lipidome dataset of 80 and 79 PDAC patients from an identification (ID) and validation 1 (VD1) cohort [12], and included further metabolome and lipidome plasma data from a second validation cohort (VD2) containing 202 PDAC patients (unpublished data). With different mass spectrometry approaches, we detected in each PDAC patient cohort (ID, VD1 and VD2) more than 660 metabolites in the plasma of these patients (Figure 1A,B). Metabolites with more than 10% of missing values were excluded from the analysis, which resulted in an overall detection of 589, 600 and 624 metabolites in ID, VD1 and VD2, respectively. Compiling the three patient cohorts, 497 metabolites were present in all three cohorts and they were used for further non-negative matrix factorization (NMF) analysis (Figure 1B). Due to the combination of global and, particularly, lipid metabolome profiling, more than 76% of the 497 metabolites belonged to the class of complex lipids and fatty acids, while amino acids and amino acid-related metabolites were the second most abundant metabolites measured in our data set (Figure 1C). Notably, complex lipids could be further subclassified into multiple lipid families. Sphingomyelins, ceramides, triacylglycerol and phosphatidylcholines represented the most abundant lipid classes (Figure 1D).

### 3.2. PDAC Metabolome Plasma Profiles Cluster into Three Metabolic Subtypes

**Figure 2:**
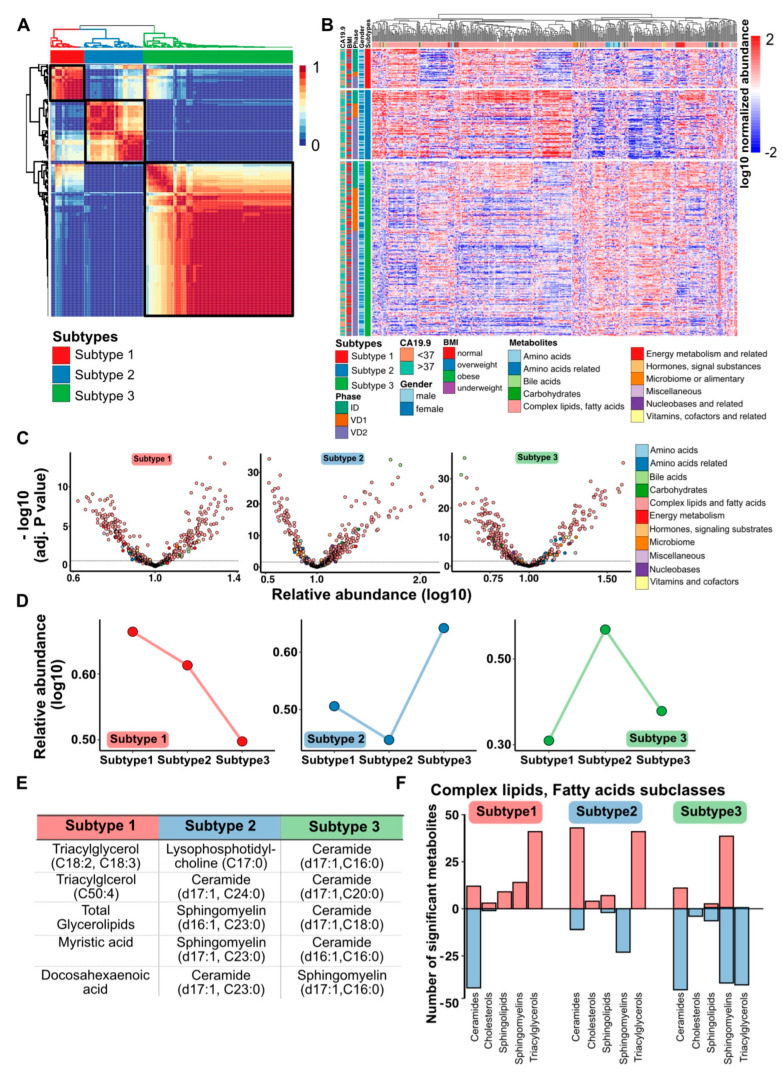
Metabolic classification of the PDAC disease cohort: (**A**) unsupervised consensus classification of PDAC lipidomics data representing 361 patients using non-negative matrix factorization (NMF). (**B**) Heatmap of three consensus clusters based on plasma metabolome profiling. Data are shown as log10 normalized abundance of the metabolites. (**C**) Distribution of detected metabolites according to *p*-value in each subtype. Relative abundance is normalized by log10. (**D**) Median differential distributions of top 30 identified metabolites from each subtype. (**E**) Table shows a list of top 5 discriminative metabolites from each subtype. (**F**) Bar graph exhibits the 5 prominent subclasses from complex lipid and fatty acid classes, with significant fold changes per subtype compared to the rest. Red bar depicts upregulated metabolites, whereas blue bar denotes downregulated metabolites.

To investigate whether the combined global and lipid metabolome plasma profiles of the 361 PDAC patients clustered into distinct metabolic phenotypes, we performed an unsupervised NMF cluster analysis [18]. The best NMF partitioning revealed three distinct metabolome subtypes, including 50 patients in subtype 1, 88 patients in subtype 2 and 223 patients in subtype 3 (Figure 2A). Plotting the log10-transformed metabolite levels of all 497 metabolites according to the subtypes, we detected distinct patterns of metabolites for each metabolic PDAC subtype. No correlation to CA19-9 levels, BMI, study phase cohort or gender was detected, ruling out bias towards general body conditions, gender-dependent metabolic changes or a pre-analytical bias due to blood collection, sample age or storage at different study phases (ID, VD1 and VD2) (Figure 2B). Each metabolic PDAC subtype exhibited significant different metabolites in comparison to all other patients, and with respect to all metabolic classes (Figure 2C). From these significantly changed metabolites, we selected classifier metabolites, which differentiated best between the subtypes. The relative abundance of the top 30 classifier metabolites demonstrated that metabolites associated with subtype 1 were highly enriched in subtype 1, whereas the metabolites showed an intermediate or low level in subtype 2 and 3, respectively (Figure 2D, left panel). Interestingly, metabolites defining subtype 2 and subtype 3 were negatively correlated. The top 30 classifier metabolites representing subtype 2 exhibited a reduced presence in subtype 2 and a strong enrichment in subtype 3, whereas metabolites representing subtype 3 showed an overall reduced presence in subtypes 1 and 3 and a high abundance in subtype 2 (Figure 2D, middle and right panel). To attain an impression on the involved metabolites, we listed the top 5 metabolites defining each subtype. Subtype 1 was associated with glycerol lipids; subtype 2 was represented by a loss of long-chain ceramides and sphingomyelins, whereas subtype 3 showed a reduced relative abundance of short-chain ceramides and sphingomyelin (Figure 2E). Interestingly, all the top 5 metabolites belonged to the class of complex lipids and fatty acids. Thus, we determined the distribution of specific lipid and fatty acid subclasses for each PDAC subtype and uncovered that subtype 1 was associated with a loss of ceramides and a strong enrichment of triacylglycerols. In contrast, subtype 2 revealed a high abundance of ceramides and triacylglycerol and reduced levels of sphingomyelins, while subtype 3 was characterized by a huge reduction in ceramide and triacylglycerol levels, as well as up- and downregulated levels of sphingomyelin species (Figure 2F). In summary, the abundance and composition of complex lipids in blood plasma of PDAC patients define specific metabolic tumor subtypes.

### 3.3. Sphingolipid-Related Pathways Differ Most between Different Metabolic PDAC Subtypes

**Figure 3:**
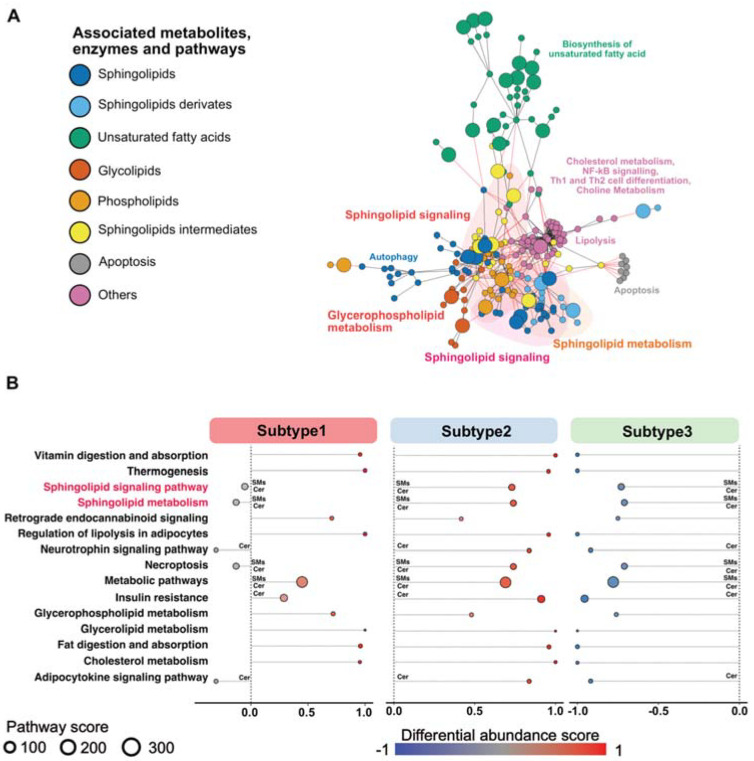
Differential metabolite abundance of complex lipid and fatty acids in metabolic subtypes: (**A**) network analysis depicts significantly enriched metabolites and pathways. Different node colors indicate different network clusters or closely interconnected metabolites. Red lines represent interacting nodes. (**B**) A pathway-based analysis of significant metabolic changes comparing patients assigned to a specific metabolic subtype to the rest of patients. The differential abundance score captures the average gross changes for all metabolites in a pathway. A score of 1 indicates increase in all measured metabolites in the pathway, whereas −1 indicates decrease in all measured metabolites in a pathway. Pathways associated with sphingomyelins and ceramides are marked with SMs/Cer.

To identify whether global metabolic networks and pathways are linked to changes in metabolite levels, we performed a KEGG annotation analysis of the differentially abundant metabolites in each subtype and plotted their connections (Figure 3A). The highly abundant sphingolipids, glycolipids and phospholipids, as well as metabolites involved in lipolysis, cholesterol and choline metabolisms, were placed in the center of the network analysis, which indicates that they are functionally related to each other (Figure 3A). Separated pathways contained metabolites that played a role in the biosynthesis of unsaturated fatty acids, apoptosis and autophagy (Figure 3A). To capture the significant metabolic changes that are linked to up- or downregulated pathways for each PDAC subtype, we calculated differential abundance scores (DAS) of the annotated metabolic pathways (Figure 3B). Overall, we uncovered that subtype 1 showed a mixed phenotype, represented by up- and downregulated metabolic pathways. In contrast, subtype 2 demonstrated a strong enrichment and subtype 3 showed a strong reduction in the associated pathways identified by our metabolic network analysis. Particularly, the KEGG pathways, “sphingolipid signaling pathway” and “sphingolipid metabolism”, revealed a distinguishable pattern between the different subtypes (Figure 3B), which suggests an important function of sphingolipids in defining metabolic PDAC cancer subtypes.

### 3.4. PDAC Subtypes Differ in Their Metabolic Programs

**Figure 4:**
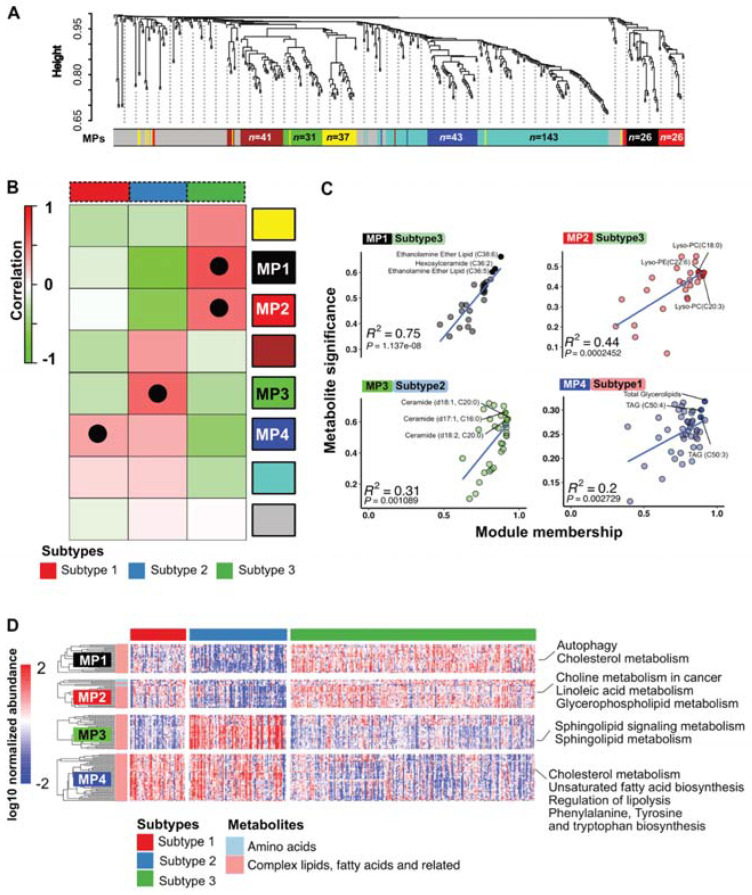
Core metabolic programs (MPs) define metabolic subtypes: (**A**) WGCNA cluster dendrogram of differentially expressed metabolites. Each leaf (vertical line) in the dendrogram corresponds to a metabolite. The colored row underneath the dendrogram shows the assigned modules, called metabolic programs (MPs). (**B**) Heatmap of metabolic programs (MPs) that were correlated to metabolic PDAC subclasses. Black dots denote metabolic networks with highest significance for a PDAC subclass. (**C**) Scatter plot depicts correlations between enriched metabolic programs (MPs) and metabolic subtype. The three most significant metabolites are named. Pearson correlation coefficient and *p*-value are presented in the plot. *p*-value < 0.05 is considered significant. (**D**) Heatmap represents differential pattern of log10 normalized metabolites abundance in the specified MPs that showed the highest correlation to the metabolic subtypes.

To further analyze the metabolic reprogramming of PDAC and to uncover specific metabolic processes that are important in PDAC subtypes, we performed a weighted correlation network analysis (WGCNA) to detect clusters of correlated genes that are associated with biological functions or pathways. Overall, the hierarchical clustering of all measured metabolites revealed distinct modules, which merge into seven clusters with similar profiles that we defined as metabolic programs (MPs). Each module contained a specific number of metabolites (see Figure 4A). Metabolites that were not assigned to any program were compiled in the grey module (Figure 4A). We correlated the MPs to the PDAC subtypes and detected several significant positive correlations between a module membership score that determines the cluster affiliation of specific metabolites and their significant enrichment in the PDAC subtypes (Figure 4B). For each metabolic subtype, we depicted the MPs with the highest correlation to a specific metabolic PDAC subtype (Figure 4C). MP1 (black) and MP2 (red) showed a very strong positive correlation with PDAC subtype 3, whereas MP3 (green) was highly associated with PDAC subtype 2. MP4 (blue) demonstrated a significant positive correlation with PDAC subtype 1 (Figure 4C). To uncover candidates that might drive the metabolic reprogramming into three different PDAC subtypes, we displayed the top 3 key metabolites in our correlation analysis. Interestingly, PDAC subtype 1 relied on triacylglycerols, subtype 2 on ceramides and subtype 3 on ethanolamine ether lipids and lyso-phosphatidylcholine (Figure 4C), which corroborated our findings of the PDAC subtype classifier genes (identified in Figure 2D,E). Next, we plotted the normalized abundance of the metabolites belonging to the four most relevant MPs for each PDAC subtype and performed KEGG annotation (Figure 4D). Overall, we detected complex lipids as the major components defining the MPs, except for MP2, which contained amino acids in addition (Figure 4D). Notably, MP1 and MP2, which are highly associated with PDAC subtype 3, were functionally correlated with the KEGG pathways’ autophagy, cholesterol metabolism, choline metabolism in cancer, as well as linoleic acid and glycerophospholipid metabolism. The MP3 and the associated PDAC subtype 2 relied functionally on sphingolipid signaling and sphingolipid metabolism. In addition, MP4, which was highly correlated to PDAC subtype 1, revealed a functional rewiring of the cholesterol metabolism, unsaturated fatty acid biosynthesis and regulation of lipolysis (Figure 4D). Hence, each metabolic PDAC subtype exhibits different lipid signatures that can be identified in the plasma metabolome.

### 3.5. Plasma Metabolic PDAC Subtypes Do Not Overlap with Molecular PDAC Subtypes

**Figure 5:**
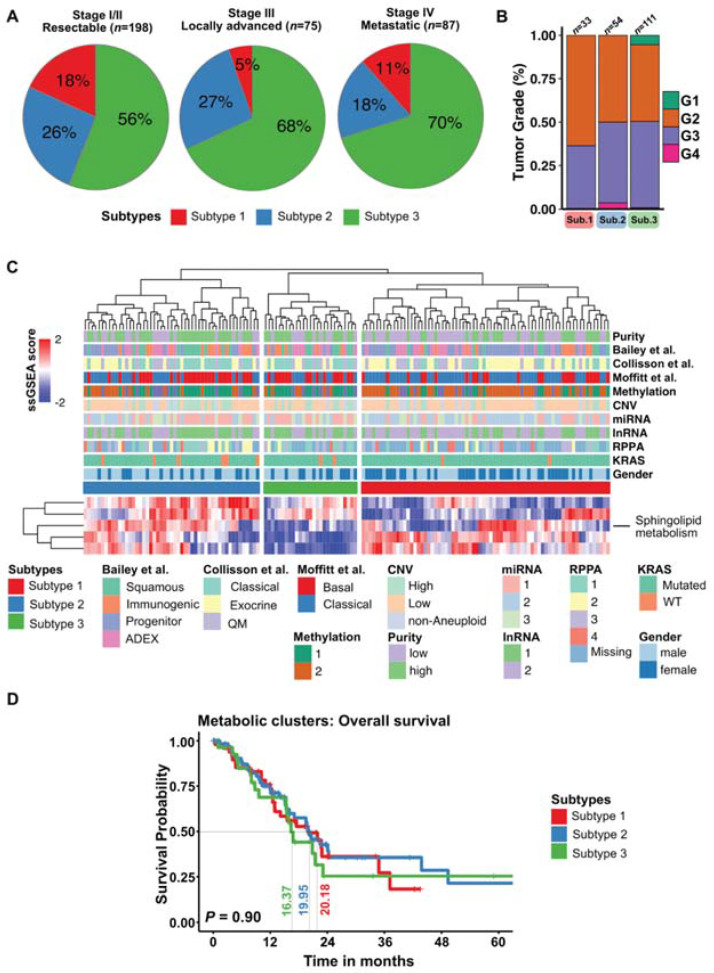
Correlation between TCGA-PAAD transcriptome and metabolic subtypes: (**A**) pie charts show proportions of metabolic subtypes in each clinical stage of PDAC. (**B**) Stacked bar plot of tumor grades for each metabolic subtype. (**C**) Consensus clustering heatmap was performed on metabolic genes associated with identified pathways in metabolite subtypes from the TCGA-PAAD data set. Clinical characteristics and transcriptomic subtypes associated with TCGA-PAAD were marked with color bars above heatmap. (**D**) Kaplan–Meier curves of cancer specific overall survival of individual subtypes delineated from TCGA-PAAD dataset. *p* < 0.05 is considered significant.

To address the biological and clinical relevance of metabolic PDAC subtypes, we investigated the distribution of the metabolic subtypes in tumor stage and grade. The collected plasma samples stemmed from 198 patients with stage I/II, from 75 patients with locally advanced stage III and from 87 patients in stage IV representing metastatic cases (Figure 5A). Interestingly, we identified a higher number of patients belonging to the metabolic PDAC subtype 1 in stage I/II PDAC tumor samples. In contrast, PDAC patients with locally advanced stage III, as well as metastatic stage IV tumors, were associated more often with a metabolic PDAC subtype 3 (Figure 5A). However, we did not observe any association of the metabolic PDAC subtypes with tumor grade, which might be due to incomplete grading information available for only 198 patients (Figure 5B). To compare the metabolic tumor subtypes with the recently described molecular PDAC subtypes, we extracted the gene expression data of KEGG pathway components that were related to the PDAC subtypes (Figure 3B) and the associated metabolic programs (Figure 4D) from the TCGA-PAAD dataset [22,23]. We performed single-sample GSEA (ssGSEA) and merged the normalized gene expression data of the KEGG pathway components into enrichment scores for each patient sample. We plotted the data in a heatmap that represented biological activity levels of the indicated KEGG pathways (Figure 5C). Notably, the activity of the sphingolipid metabolism pathway genes resembled the metabolic phenotypes well. We detected a cluster with a mixed pattern, representing subtype 1, as well as two clusters with enriched and decreased sphingolipid metabolism activity, representing subtypes 2 and 3, respectively (Figure 5C). However, the metabolic PDAC subtype clusters that were based on transcriptomic data of complex lipid metabolism pathway genes revealed no overlap with the recently identified molecular PDAC subtypes described by Collisson et al., Moffitt et al. or Bailey et al. [4,5,6]. Moreover, we did not detect an association of the metabolic PDAC subtypes with tumor cell purity, DNA methylation, CNV, miRNA, lnRNA or RPPA sub-classes. KRAS wildtype and male/female cases were also equally distributed in the transcriptomic metabolic subclasses (Figure 5C). Of note, from the TCGA data set, we were able to investigate the overall survival probabilities for each metabolic PDAC subtype and detect that metabolic PDAC subtype 3 showed the worst median survival rate of 16.37 months, whereas PDAC subtypes 1 and 2 exhibited median survival times of 19.95 and 20.18 months, respectively.

## 4. Discussion

To reveal biological heterogeneity of pancreatic cancer, previous studies focused on gene mutations or changes in gene expression to stratify PDAC patients into molecular subtypes [4,5,6,24]. The various research efforts on transcriptome data of PDAC samples reached more or less a consensus on two major molecular PDAC types, described as classical and basal-like tumor subtypes [10]. However, most of the studies were performed on small single-tissue pieces from resected tumor specimens reflecting the molecular classification of PDAC at a resectable stage, which represents only a minority of PDAC cases [2]. Interestingly, a recent study from Chan-Seng-Yue et al. on single-cell resolution uncovered that classical and basal-like molecular programs exist side-by-side in advanced PDAC cases, revealing high intratumoral heterogeneity [8]. Thus, determining molecular tumor subclasses from one piece of tumor tissue collected from resected specimens or core needle biopsy might not demonstrate the full spectrum of molecular changes in PDACs. The fact that metabolic rewiring of cancer cells can be measured in the blood of cancer patients, independent of molecular tumor tissue heterogeneity, prompted us to investigate metabolic programs in blood samples of PDAC patients. From an untargeted metabolic profiling of 361 PDAC blood plasma samples, which primarily focused on complex lipids, we identified three metabolic PDAC subclasses showing major differences in triacylglycerol, ceramide and sphingolipid metabolism. To the best of our knowledge, our approach uncovered, for the first time, metabolic PDAC subtypes that were detectable in blood plasma samples of PDAC patients. Although plasma samples are an easily accessible tool, further investigations are needed to clarify whether metabolic signatures are derived from one predominant tumor cell type or from a minor tumor cell population with high metabolic activity, or even from a composition of all tumor cells. Previous studies on metabolic PDAC subtypes used either transcriptome data of differentially expressed metabolic pathway genes in tumor tissue samples [14], or performed a metabolic profiling on primary PDAC cell lines [13] A study by Daemen et al. found three metabolic PDAC subtypes based on metabolome profiles of PDAC cell lines. They mainly describe a glycolytic subtype that is associated with a more aggressive mesenchymal PDAC phenotype using glucose for glycolysis and lactate generation, as well as a lipogenic subtype that is associated with a more differentiated tumor phenotype that metabolizes glucose primarily for de novo lipogenesis [13]. In contrast, Karasinska et al. focused on differential expression of genes involved in glycolysis and cholesterol biosynthesis in PDAC tissue samples [14]. They defined four metabolic PDAC clusters, with a quiescent subtype showing decreased expression of glycolysis and cholesterol biosynthesis genes, a glycolytic and cholesterogenic phenotype demonstrating increased expression of glycolysis and cholesterol biosynthesis genes, respectively, as well as mixed subtype displaying active gene expression of both profiles. As previously described, the glycolytic PDAC subtype was associated with the more aggressive basal-like phenotype and a worse prognosis, whereas the cholesterogenic cluster was related to the classical PDAC subtype [5,6,9]. In our study, we identified three metabolic PDAC subtypes that showed distinct patterns of ceramides and sphingomyelin metabolites, as well as other complex lipid components, in blood plasma samples. Subtype 3 demonstrated a reduced abundance of ceramides, sphingomyelin and other sphingolipids, whereas subtype 2 showed very high levels of these metabolites. Interestingly, a reduced expression of related pathway genes in tumor tissue was associated with the lowest survival rate. However, our metabolic PDAC subtypes did not show any correlation with the described molecular PDAC subtypes [5,6,9,24]. Further experimental studies on these low-sphingolipid and high-sphingolipid PDAC subtypes are necessary to assess their characteristics and tumorigenic features. The sphingolipid metabolism and, particularly, the abundance of fatty acids with different chain lengths have context and cell type-specific functions on cell death and cell survival [25]. The impact of sphingolipid metabolism on pancreatic tumor growth and tumor phenotypes is not well described. In the context of membrane lipids, sphingolipids, which reside extensively on the outer leaflet of the membrane, regulate membrane fluidity, which is important for membrane plasticity during vesiculation, as well as endo/exocytosis [26,27,28]. In addition, membrane proteins and associated membrane signaling components compartmentalize within membrane domains at steady-state conditions or in response to stimulation or metabolic signals. These detergent-resistant, ordered but dynamic lateral domains within plasma membranes are enriched with sphingomyelins and cholesterol [29,30,31], and are termed “lipid rafts” [32]. Their composition can be dynamically altered upon intracellular stress signals or receptor ligation [32]. Due to their dynamic nature, lipid rafts and microdomains regulate cell adhesion and membrane signaling through proteins within lipid rafts. Non-constitutive protein components of the rafts fluctuate dramatically in the membranes of cancer cells, which impacts on cell proliferation, signaling, protein trafficking, adhesion and apoptosis [33]. It is well known that sphingomyelin-enriched lipid raft microdomains are preferred substrates for acid sphingomyelinase, an enzyme that hydrolyzes sphingomyelins to ceramides. These ceramide microdomains are involved in signal transduction. Thus, plasma membrane levels of sphingomyelins maintain the intricate balance between signal transduction and membrane biophysics through the changes in lipid membrane composition as a result of the sphingomyelin pathway [34]. It has been shown that elevated C16 and C24:1 ceramide in PDAC tissue and increased glycosylated ceramides can be detected in serum samples of patients with lymph node metastases [35], suggesting that specific ceramides promote metastasis formation. Experimental data support this notion. An increased ceramide conversion into ceramide-1-phosphate (C1P) was shown to play a role in cancer stem cell migration, facilitating PDAC tumor growth [36]; however, C1P inhibition reduced the motility of PDAC cell lines [37]. Other lipid metabolites, such as cholesterol, were described to regulate PDAC differentiation. Other than the correlation with reduced cholesterol levels in basal-like PDAC subtypes, a sophisticated study of Gabitova-Cornell et al. demonstrated that inhibition of cholesterol biosynthesis drives the epithelial-to-mesenchymal transition (EMT) of pancreatic tumor cells and the establishment of a more aggressive tumor phenotype [38]. Though it has been shown that sphingolipids, including the two central bioactive lipids, ceramide and sphingosine-1-phosphate (S1P), have opposing roles in regulating cancer cell death and survival by evading chemoresistance [39], cellular and molecular determinants regulating the selectivity of one sphingolipid metabolite over another are poorly understood. Sphingolipids are known to be involved in apoptosis and cell survival mechanisms. Recent findings suggest ceramide as a critical mediator of cell death, whereas sphingosine-1-phosphate and glucosylceramide act as pro-survivals signals. Moreover, augmented levels of modified species of gangliosides (complex glycosphingolipids) were related to anti-apoptotic roles and they increased in the expression of multidrug resistance gene MDR1. The fact that the mechanism of action of chemotherapies leads to apoptosis in cancer cells suggests an important role for sphingolipid in chemotherapy resistance [40]. It was shown that the intrinsic cellular ceramide/S1P ratio could act as a predicting marker for pancreatic cancer cell resistance to the chemotherapeutic drug gemcitabine, where increasing this ratio was found to optimize cell sensitivity [39]. Nevertheless, cellular and molecular determinants regulating selectivity for one sphingolipid metabolite over another are poorly understood. Subjected to prospective validation and mechanistic elucidation, the identified subtypes can be translated for treatment stratification and as predictive biomarkers. In the future, further studies are needed to address the contribution of complex lipids, such as sphingolipids or cholesterol, to cancer growth and metastasis formation. It is important to understand how metabolites control cancer characteristics and whether they might serve as potential targets for new therapeutic options. Our findings of a sphingolipid-low and -high PDAC subtype, detected in blood plasma samples, pave the way for further studies to investigate sphingolipid metabolisms in pancreatic cancer processes.

## Data Availability

All data and analyses scripts will be available upon request.

## Supplementary Materials

The following are available online at https://www.mdpi.com/article/10.3390/cells10071821/s1, Table S1: Demographics and distribution of clinical characterization according to project phases.
