# Supplementary material for: Plasma Metabolome Profiling Identifies Metabolic Subtypes of Pancreatic Ductal Adenocarcinoma
Source: Cells. 2021 Jul 19;10(7):1821. doi: 10.3390/cells10071821 (PMC8305839; doi:10.3390/cells10071821)
Supplement: Supplementary file 1 [file cells-10-01821-s001.zip › cells-1273025-supplementary.pdf]

Supplementary Table S1: Demographics and distribution of clinical characterization according to project phases.

	ID (N=80)	VD1 (N=79)	VD2 (N=202)	Total (N=361)
**Center**				
Berlin-Mitte	0 (0.0%)	0 (0.0%)	2 (1.0%)	2 (0.6%)
Berlin-Virchow	0 (0.0%)	0 (0.0%)	62 (30.7%)	62 (17.2%)
Bochum	0 (0.0%)	0 (0.0%)	64 (31.7%)	64 (17.7%)
Dresden	80 (100.0%)	79 (100.0%)	74 (36.6%)	233 (64.5%)
**Gender**				
female	34 (42.5%)	42 (53.2%)	89 (44.1%)	165 (45.7%)
male	46 (57.5%)	37 (46.8%)	113 (55.9%)	196 (54.3%)
**UICC classification**				
IA	0 (0.0%)	1 (1.3%)	4 (2.0%)	5 (1.4%)
IB	2 (2.5%)	0 (0.0%)	2 (1.0%)	4 (1.1%)
IIA	16 (20.0%)	11 (13.9%)	33 (16.4%)	60 (16.7%)
IIB	37 (46.2%)	28 (35.4%)	64 (31.8%)	129 (35.8%)
III	19 (23.8%)	26 (32.9%)	30 (14.9%)	75 (20.8%)
IV	6 (7.5%)	13 (16.5%)	68 (33.8%)	87 (24.2%)
**Age**				
Mean (SD)	68.6 (7.3)	67.6 (9.4)	66.9 (9.6)	67.4 (9.1)
**Diabetic status**				
No	40 (50.0%)	43 (54.4%)	140 (69.3%)	223 (61.8%)
Yes	40 (50.0%)	36 (45.6%)	62 (30.7%)	138 (38.2%)
**BMI**				
Mean (SD)	24.7 (3.1)	25.5 (4.2)	24.6 (3.9)	24.8 (3.8)
**Tumor size (pT)**				
1	0 (0.0%)	0	6 (3.0%)	6 (2.3%)
2	4 (6.6%)	0	5 (2.5%)	9 (3.5%)
3	57 (93.4%)	0	99 (50.0%)	156 (60.2%)
4	0 (0.0%)	0	10 (5.1%)	10 (3.9%)
Inoperable	0 (0.0%)	0	78 (39.4%)	78 (30.1%)
**Lymph node Invasion (pN)**				
0	19 (31.1%)	0	44 (31.0%)	63 (31.0%)
1	42 (68.9%)	0	77 (54.2%)	119 (58.6%)
Not assessable	0 (0.0%)	0	21 (14.8%)	21 (10.3%)
**Metastases (pM)**				
0	55 (90.2%)	0	125 (62.8%)	180 (69.2%)
1	4 (6.6%)	0	67 (33.7%)	71 (27.3%)
Not assessable	2 (3.3%)	0	7 (3.5%)	9 (3.5%)
**Tumor Grade**				
1	0 (0.0%)	0	6 (3.2%)	6 (2.5%)
2	28 (54.9%)	0	69 (36.3%)	97 (40.2%)
3	23 (45.1%)	0	69 (36.3%)	92 (38.2%)
4	0 (0.0%)	0	3 (1.6%)	3 (1.2%)
Not assessable	0 (0.0%)	0	43 (22.6%)	43 (17.8%)
**CA 19.9 levels (U/ml)**				
≤37	17 (21.2%)	20 (25.3%)	58 (28.7%)	95 (26.3%)
>37	63 (78.8%)	59 (74.7%)	144 (71.3%)	266 (73.7%)
